# Genome-Wide Identification and Characterization of WD40 Protein Genes in the Silkworm, *Bombyx mori*

**DOI:** 10.3390/ijms19020527

**Published:** 2018-02-09

**Authors:** Songzhen He, Xiaoling Tong, Minjin Han, Hai Hu, Fangyin Dai

**Affiliations:** State Key Laboratory of Silkworm Genome Biology, Key Laboratory of Sericultural Biology and Genetic Breeding, Ministry of Agriculture, Southwest University, Chongqing 400715, China; szhe@swu.edu.cn (S.H.); xltong@swu.edu.cn (X.T.); minjinhan@126.com (M.H.); huhaiswu@163.com (H.H.)

**Keywords:** WD40 proteins, identification, characterization, multiple animals, silkworm

## Abstract

WD40 proteins are scaffolding molecules in protein-protein interactions and play crucial roles in fundamental biological processes. Genome-wide characterization of WD40 proteins in animals has been conducted solely in humans. We retrieved 172 WD40 protein genes in silkworm (*BmWD40s*) and identified these genes in 7 other insects, 9 vertebrates and 5 nematodes. Comparative analysis revealed that the WD40 protein gene family underwent lineage-specific expansions during animal evolution, but did not undergo significant expansion during insect evolution. The *BmWD40s* were categorized into five clusters and 12 classes according to the phylogenetic classification and their domain architectures, respectively. Sequence analyses indicated that tandem and segmental duplication played minor roles in producing the current number of *BmWD40s*, and domain recombination events of multi-domain *BmWD40s* might have occurred mainly after gene duplication events. Gene Ontology (GO) analysis revealed that a higher proportion of *BmWD40s* was involved in processes, such as binding, transcription-regulation and cellular component biogenesis, compared to all silkworm genes annotated in GO. Microarray-based analysis demonstrated that many *BmWD40s* had tissue-specific expression and exhibited high and/or sex-related expression during metamorphosis. These findings contribute to a better understanding of the evolution of the animal WD40 protein family and assist the study of the functions of *BmWD40s*.

## 1. Introduction

WD40 proteins, also known as WD-repeat proteins or WD40-containing proteins, comprise one of the largest protein families in eukaryotes, but they are rare in prokaryotes [1,2]. WD40 protein was first identified and analyzed as a G protein β-subunit (bovine transducin β-subunit), containing repeated units of ~43 residues with conserved Glycine Histidine (GH) and tryptophan aspartate (WD) motifs [3]. Proteins with these repeated residue units (WD40 repeats or blades) were defined as WD40 proteins [1,4]. Generally, each WD40 repeat contains 40–60 residues with a WD motif and is able to fold into four anti-parallel β-strands [5]. A total of seven repeats constitute a canonical WD40 domain, which folds into a β-propeller structure [6,7,8]. Usually, a WD40 protein would contain 4–9 blades in one β-propeller [9].

WD40 proteins function as scaffolds for protein-protein interactions or provide platforms to recruit diverse molecules that form functional complexes [1,10,11]. This helps the proteins perform their bioactivities. WD40 proteins play vital roles in many basic biological processes, such as DNA replication, transcriptional regulation and damage responses [12,13,14], RNA processing and modification [15,16,17,18], protein degradation [19], histone recognition and modification [20,21], apoptosis [22,23,24] and signal transduction [25,26,27]. Many WD40 proteins are implicated in diseases. For instance, WDR36 was found to be mutated in primary open-angle glaucoma [28]; WDR45 is implicated in neurodegeneration [29]; WDR62 is associated with human microcephaly [30,31]; and FBXW7 and TLE1 are involved in tumors [32,33,34]. BmPLA2 of silkworms influences fat-body metabolism. Knockdown of the *BmPLA2* gene resulted in reduced development and death [35].

Genome-wide systematic characterization of this protein family has been conducted in many species, including rice, foxtail millet, cucumber, *Arabidopsis* and humans [2,36,37,38,39,40]. In animals, this family has been thoroughly studied in humans, but little is known about WD40 genes in other species. 

The silkworm (*Bombyx mori*) is an economically-important insect for silk production. It is also an ideal model species for microbiology, physiology, genetics and functional genomics analysis [41]. In the present study, we carried out a genome-wide identification of the WD40 protein gene family in multiple animals and performed gene expression pattern and sequence analysis of silkworm WD40 proteins. Our findings could facilitate the understanding of the WD40 protein family and provide important clues for subsequent research on its functions.

## 2. Results and Discussion

### 2.1. Identification of WD40 Proteins

To identify WD40 proteins in *Bombyx mori*, the hmmsearch program in the HMMER3.0 package (http://hmmer.org/) [42] was applied to search in the silkworm protein database using the Hidden Markov Model (HMM) profile for the WD repeat (PF00400). Redundant sequences were discarded manually. The presence of WD40 repeats was confirmed by CDD (Conserved Domain Database, http://www.ncbi.nlm.nih.gov/Structure/cdd/wrpsb.cgi) [43], SMART (Simple Modular Architecture Research Tool, http://smart.embl-heidelberg.de/) [44] and WDSP (WD40-repeat Protein Structure Predictor, http://wu.scbb.pkusz.edu.cn/wdsp/predictor.jsp) [45] search-based domain analysis. As a result, 172 non-redundant silkworm WD40 proteins (*Bm*WD40) were retrieved, and each protein represented the typical product of a gene. The 172 *BmWD40* genes were numbered from *BmWD001*–*BmWD172* based on their order on chromosomes (Table S1).

In silico analysis showed that the *Bm*WD40 proteins varied greatly in length and physicochemical properties. The lengths of *Bm*WD40s ranged from 99 residues–3007 residues. The molecular weights varied from 11.061 kDa– 30.895 kDa. Isoelectric points ranged from 4.44–9.76. The number of WD40 repeats varied from 2–21 (Table S1), suggesting the existence of multiple, incomplete or atypical WD40 domains in some proteins. A total of 64 of the 172 *Bm*WD40s were DWD (Damaged DNA binding WD40) proteins (Table S1). These proteins are a subset of WD40 proteins containing a conserved DWD box in the WD40 repeats. They modulate numerous biological processes by ubiquitin-dependent proteolysis [46,47]. Basic information of these genes is summarized in Table S1.

**Figure 1:**
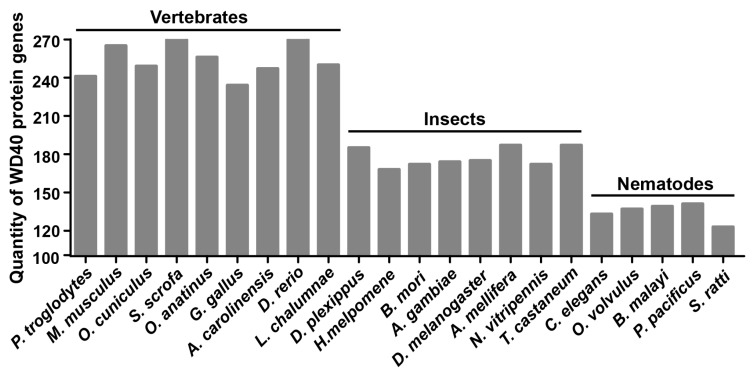
The number of WD40 protein genes in different species. WD40 protein genes in 8 insects, 9 vertebrates and 5 nematodes were identified. The total numbers of WD40 protein genes present in the genomes of the eight insects were similar. The number of WD40 protein genes in these insects (mean ~178) is larger than that in nematodes (mean ~135) and smaller than that in vertebrates (mean ~256).

WD40 protein genes in 7 other insect species, as well as 9 vertebrates and 5 nematodes were identified using the same approach, and the WD40 protein genes are listed in Table S2. The total numbers of WD40 protein genes present in the genomes of silkworm and seven other insect species were similar (Figure 1 and Table S2), indicating that the WD40 protein gene family did not undergo significant expansion during insect evolution. However, the number of WD40 protein genes in these surveyed insects (mean ~178) is larger than the number in nematodes (mean ~135) and smaller than that in vertebrates (mean ~256) (Figure 1 and Table S2). This suggests that the WD40 protein genes have undergone a lineage-specific expansion in the more highly-evolved groups of animals compared with the lower animals. Since WD40 proteins are pivotal regulatory proteins involved in a variety of physiological processes, we speculate that lineage-specific expansion of the animal WD40 gene family evolved to meet the more complex functional requirements of highly-evolved animals.

### 2.2. Chromosomal Distribution and Gene Structure of the BmWD40 Family

**Figure 2:**
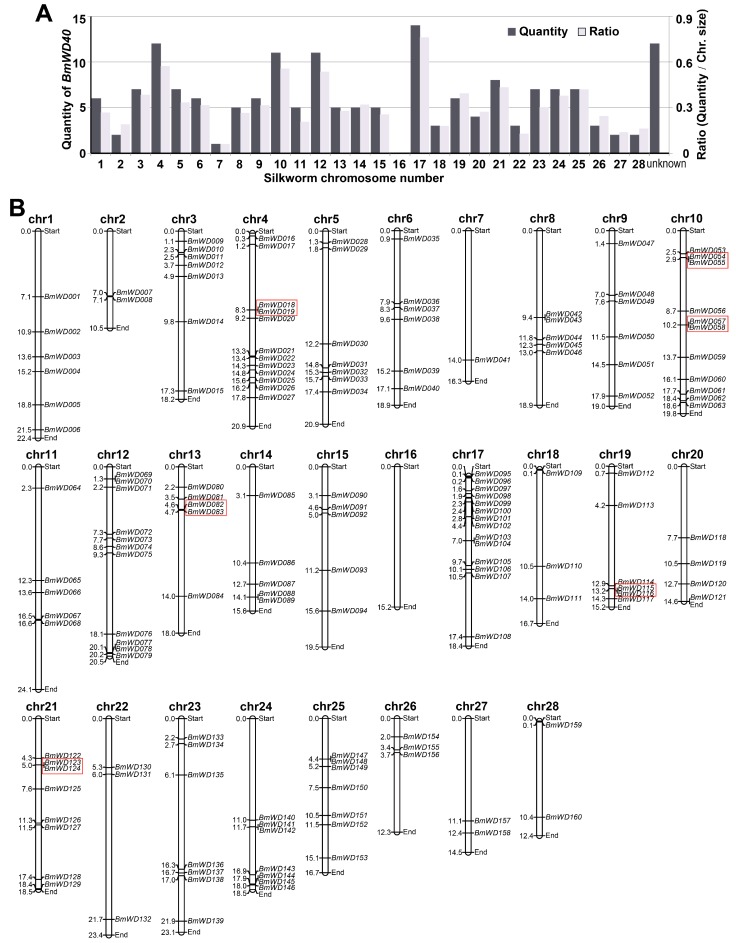
Distribution of the *BmWD40* genes on chromosomes. (**A**) Number of *BmWD40s* on each silkworm chromosome and the ratio of gene number to chromosome size to show the distribution abundance; (**B**) “*BmWD40s* distribution map” on silkworm chromosomes. Tandem duplicated genes are marked by red boxes. The chr represents chromosome. Chromosomal distances are given in Mb (Mega base).

To determine the distribution and contexts of the *BmWD40* genes family on silkworm chromosomes, a “*BmWD40s* distribution map” was constructed based on the chromosomal coordinates extracted from the silkworm genome database (http://sgp.dna.affrc.go.jp/KAIKObase/). We found that the distribution of *BmWD40* genes is widespread and uneven on the chromosomes. As shown in the “*BmWD40s* distribution map”, 160 *Bm*WD40s were assigned to 27 silkworm chromosomes (excluding chromosome 16) (Figure 2 and Table S1), while the other 12 *BmWD40s* did not have location information on chromosomes. Chromosome 17 has the largest number of *BmWD40* genes (14 genes; the ratio of gene quantity to chromosome size is 0.76), followed by chromosomes 4 (12 genes, ratio = 0.57), while lesser numbers of genes were found on chromosome 7 (two genes, ratio = 0.06) (Figure 2). The distribution of *BmWD40* genes exhibits a positional accumulation pattern on certain chromosomes. Relatively high densities of *BmWD40* genes were observed at the top of chromosomes 3, 13 and 17, as well as at the bottom of chromosomes 4, 19 and 24. Relatively low densities of *BmWD40* genes were also found in some other chromosomal regions, including the top of chromosomes 1, 2, 7, 8, 20, 24 and 27 and at the bottom of chromosomes 8, 11 and 26 (Figure 2). Previous studies have shown that the distributions of the WD40 proteins genes on chromosomes in plants and human were also extensive and uneven [37,38,39,40]. These findings suggest that the pervasive-uneven distribution is a universal characteristic of the WD40 gene family in plants and animals.

Genome duplication events, including tandem duplication and segmental duplication, are thought to contribute in the evolution and expansion of gene families [48,49]. Previous studies indicated that the expansion and distribution pattern of the WD40 gene family in *Arabidopsis* [38], rice [37] and foxtail millet [39] were due to both tandem duplication and segmental genome duplication events. Zou et al. speculated that this distribution pattern of WD40 proteins genes in humans was related to different degrees of segmental duplication on different chromosomes [40]. However, cucumber studies revealed that tandem duplication and segmental duplication did not play critical roles in the expansion of cucumber WD40 genes [38], due to the absence of the recent whole-genome duplication events and tandem duplications [50]. There is no evidence of whole genome duplication events in the silkworm. In this study, 12 (~7%) *BmWD40* genes were identified to be tandem repeats (tandem duplication of genes) based on sequence alignment and chromosomal distribution (Figure 2), and only two *BmWD40* genes (*BmWD005* and *BmWD101*) were related to potential segmental duplication events. These data suggest that tandem and segmental duplication played a minor role in the number of *BmWD40* genes. Additional study is needed to determine the mechanism behind the expansion and distribution pattern of the *BmWD40* gene family.

We also investigated gene structures and found that the intron sequences within the Open Reading Frame (ORF) of the *BmWD40* genes varied greatly and the number of introns ranged from 0–56. The longest *BmWD40* gene was *BmWD11* with about a 103.6-kb genomic sequence, while the shortest one was only 417 bp (*BmWD064*) (Table S1).

### 2.3. Phylogenetic Classification and Domain Architectures of BmWD40s

**Figure 3:**
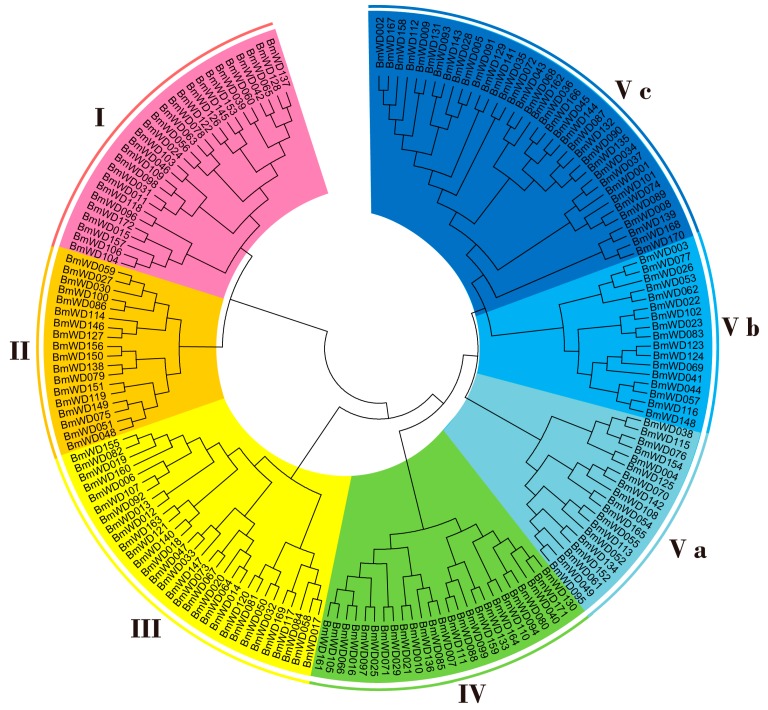
Phylogenetic classification of *Bm*WD40 proteins. MEGA6 (Molecular Evolutionary Genetics Analysis version 6.0, http://www.megasoftware.net/) was used to construct the phylogenetic tree using the neighbor-joining method with 1000 bootstrap replicates. The tree was divided into five main distinct clusters (Cluster I–V), and Cluster V was divided into three sub-clusters (Clusters Va, Vb and Vc).

To study the evolutionary relationships of the WD40 family in silkworm, 172 identified *Bm*WD40 protein sequences were used to construct a phylogenetic tree according to the Neighbor-Joining (NJ) method [51]. As shown in Figure 3, the *Bm*WD40s were categorized into five main distinct clusters (Clusters I–V) containing 27, 18, 30, 25 and 72 proteins, respectively. There were three sub-clusters (Clusters Va, Vb and Vc) in Cluster V, suggesting the complex and differentiated structure of *Bm*WD40s.

**Figure 4:**
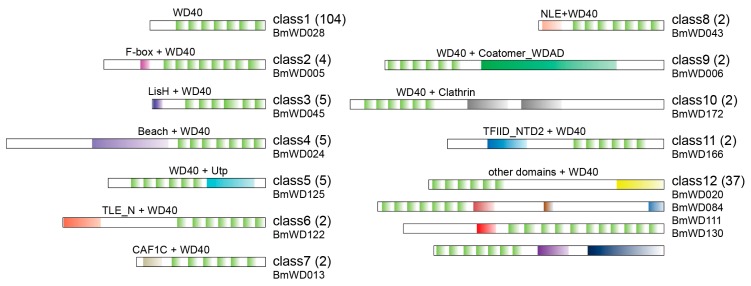
Domain architectures of *Bm*WD40 proteins. The protein structure is identified by WDSP, SMART and CDD. This organizes the *Bm*WD40s into 12 classes. The WD40 repeats are colored in light green, and other domains are filled in with other colors separately. The number of members in each class is shown in parentheses. The name of the displayed protein is given below.

To get more details of the complex protein domain structures, the domain architectures of the *Bm*WD40s were annotated. The *Bm*WD40s were categorized into 12 classes based on their domain compositions (Figure 4 and Table S1). A total of 104 *Bm*WD40s with only the WD40 domain were grouped into Class 1. The other *Bm*WD40s with additional functional domains were categorized into Classes 2–12 as follows: 4 *Bm*WD40s containing the F-BOX and U-BOX domain were grouped into Class 2; 5 *Bm*WD40s comprising the LisH domain were identified as Class 3; 5 *Bm*WD40s with the Beige/BEACH domain were categorized into Class 4; 5 *Bm*WD40s with the Utp domain were identified as Class 5; *Bm*WD40s with the TLE_N domain, subunit C of the CAF1 complex domain, the NLE (NUC) domain, the coatomer WD-associated region, the clathrin domain and the TFIID_NTD2 domain were categorized into Classes 6–11, respectively. For simplicity, the remaining 37 *Bm*WD40s with only one member for each domain-architecture were put into Class 12 (Figure 4 and Table S1). Apart from the domain architectures conserved between animal and plants, we also found potential animal-specific architectures according to previous reports [40], including Class 6 (TLE_N + WD40) and four architectures in Class 12 (Dynein_IC2 + WD40, striatin + WD40, HELP + WD40, NACHT + WD40) (Figure 4 and Table S1).

**Figure 5:**
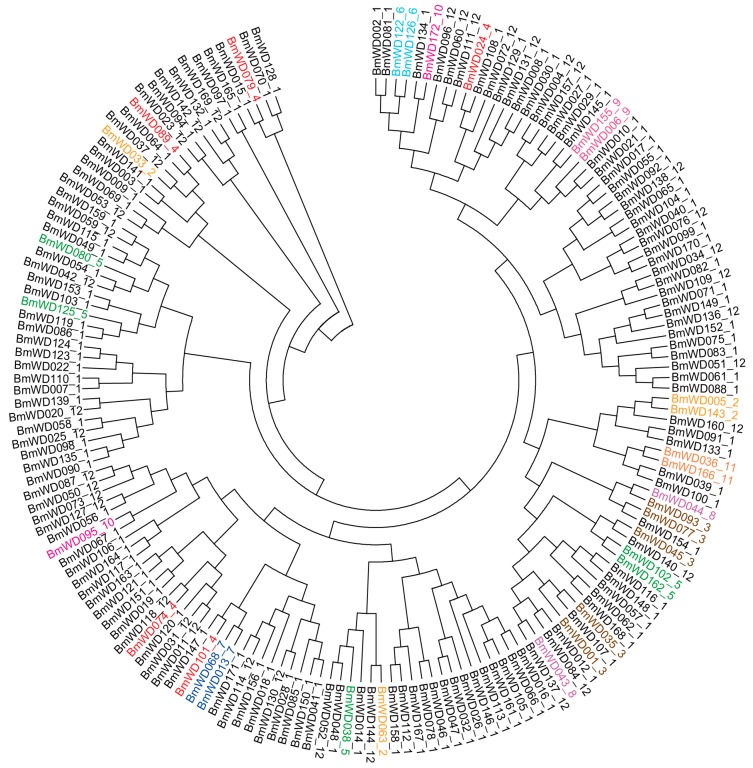
WD40 domain sequence-based phylogenetic tree of *Bm*WD40 proteins. MEGA6 was used to construct the phylogenetic tree using the neighbor-joining method with 1000 bootstrap replicates. Multi-domain *Bm*WD40 proteins from each class (i.e., from Class 2–Class 11) with multiple members for each domain-architecture are indicated by the same colors, respectively.

Domain recombination events and gene duplication events might have happened in the evolution of multi-domain protein gene families. Determining the order of these two events will promote our understanding of the evolutionary history of the gene families. Accordingly, we used the amino acid sequences of WD40 domains from the identified *Bm*WD40 proteins to construct a phylogenetic tree. Figure 5 shows that many members of Class 1 (with only the WD40 domain) are separately grouped with the members of other classes (with multi-domains) in the phylogenetic tree, and only a few multi-domain *Bm*WD40 proteins from the same classes (Class 2, 5–7 and 11) group together into one sub-clade. These results suggest that the multi-domain *Bm*WD40 protein genes may have evolved from the genes with only the WD40 domain within the same sub-clade. The domain recombination events might have happened mainly after the gene duplication events in the evolution of the multi-domain *Bm*WD40 protein genes.

### 2.4. Gene Ontology and KEGG Analysis of the BmWD40s

**Figure 6:**
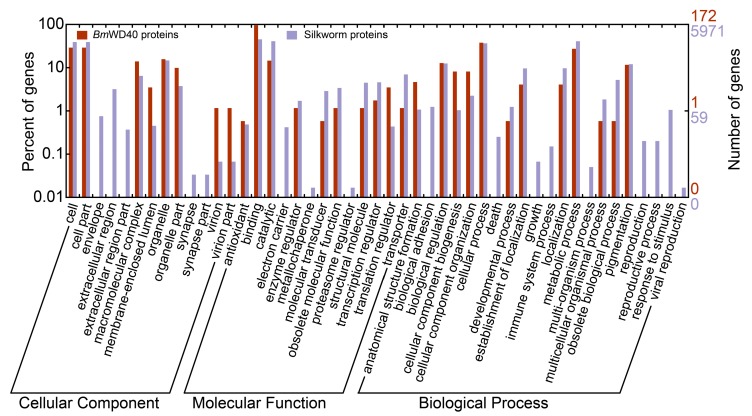
Gene Ontology (GO) categories of *BmWD40* genes and genes with GO annotations obtained from SilkDB. This analysis was visualized with WEGO (Web Gene Ontology Annotation Plotting, http://wego.genomics.org.cn/). Details are provided in Supplementary File Table S3.

To survey the functions of the *BmWD40* genes, Gene Ontology (GO) annotation was performed using Blast2GO software and also obtained from two silkworm genome databases (http://www.silkdb.org and http://sgp.dna.affrc.go.jp/KAIKObase/). Compared to a wide range of silkworm genes annotated in GO, a higher proportion of *BmWD40s* played roles in binding, transcription regulator activity, anatomical structure formation, cellular component organization and cellular component biogenesis (Figure 6). Consistent with the essential protein roles, the analysis revealed that most *BmWD40s* (165, ~95.9%) participated in binding (Figure 6, Table S3). The results also showed that the *BmWD40s* were involved in diverse biological processes and predominantly participated in cellular metabolic processes (42, ~24.4%), followed by primary metabolic processes (39, ~22.7%) and macromolecule metabolic processes (35, ~20.3%) (Table S3).

To ascertain whether the *BmWD40s* categorized into the five distinct clusters (Cluster I–V) of the phylogenetic tree had functional preference, respectively, GO analysis was also performed with the genes of the five clusters. The results revealed that the *BmWD40s* of the five distinct clusters had a variety of biological functions and were involved in various physiological processes with a degree of functional specificity. For example, the gene (*BmWD015*) participating in multicellular organismal processes and developmental processes belonged to Cluster I, and the *BmWD40s* involved in structural molecule activity (*BmWD006* and *BmWD155*) and molecular transducer activity (*BmWD014*) are only found in Cluster III. The genes implicated in transporter activity (*BmWD057* and *BmWD162*), antioxidant activity (*BmWD148*) and transcription regulator activity (*BmWD053, BmWD093* and *BmWD166*) were all grouped into Cluster V (Figure S1).

**Figure 7:**
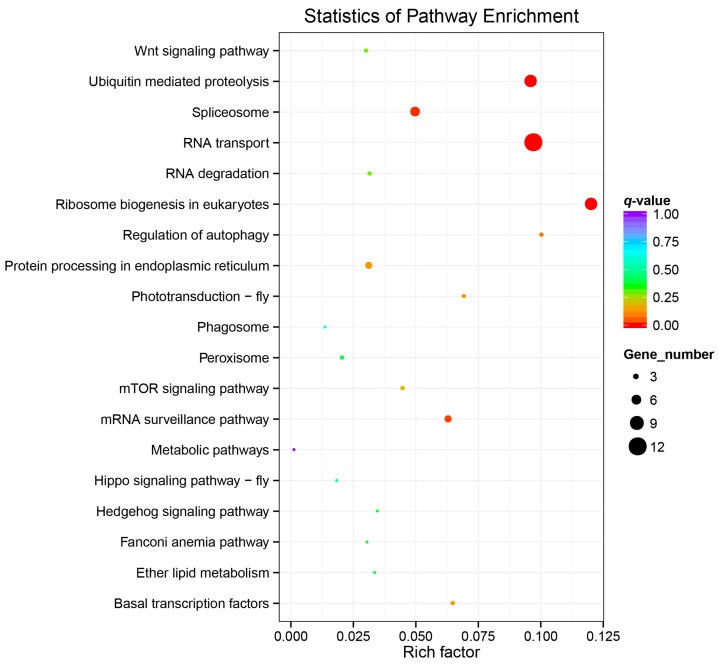
Scatterplot of Kyoto Encyclopedia of Genes and Genomes (KEGG) pathway enrichment analysis. The “Rich factor” is the ratio of the *BmWD40* gene number to the total annotated gene number in a certain pathway. The size and color of the dots represent the range of the gene number and the *q*-value, respectively. Details are provided in Supplementary File Table S4.

The *BmWD40* genes were also classified into reference pathways in the Kyoto Encyclopedia of Genes and Genomes database (KEGG, http://www.kegg.jp/) [52]. The results showed that the *BmWD40* genes were also involved in numerous biological pathways (Figure 7, Table S4). A total of seven KEGG pathways, including RNA transport (bmor03013), ribosome biogenesis in eukaryotes (bmor03008), ubiquitin-mediated proteolysis (bmor04120), spliceosome (bmor03040), mRNA surveillance pathway (bmor03015), regulation of autophagy (bmor04140) and phototransduction (bmor04745), were significantly enriched (corrected *p*-value less than 0.05) for the *BmWD40* genes.

### 2.5. Spatial and Temporal Expression Profile of the BmWD40s 

The gene expression profile can also provide a strong indication about the function and biological activity of a gene. Highly- and ubiquitously-expressed WD40 protein genes might originate early in evolution and regulate the transcription of a broad set of genes. In contrast, other genes of this family could be involved in diverse physiological processes in tissue- or stage-specific manners [39,40].

To view the spatial expression profile of *BmWD40* genes, we used the microarray gene expression dataset from SilkDB, which includes normalized gene expression levels across 10 silkworm larval tissues. Then, 130 genes were detected as expressed in at least one tissue. A total of 22 genes was expressed in all investigated tissues, and these were involved in basic molecular functions including transcription regulator, translation regulator, catalytic activity, structural molecule activity and binding (Figure S2, Table S5).

**Figure 8:**
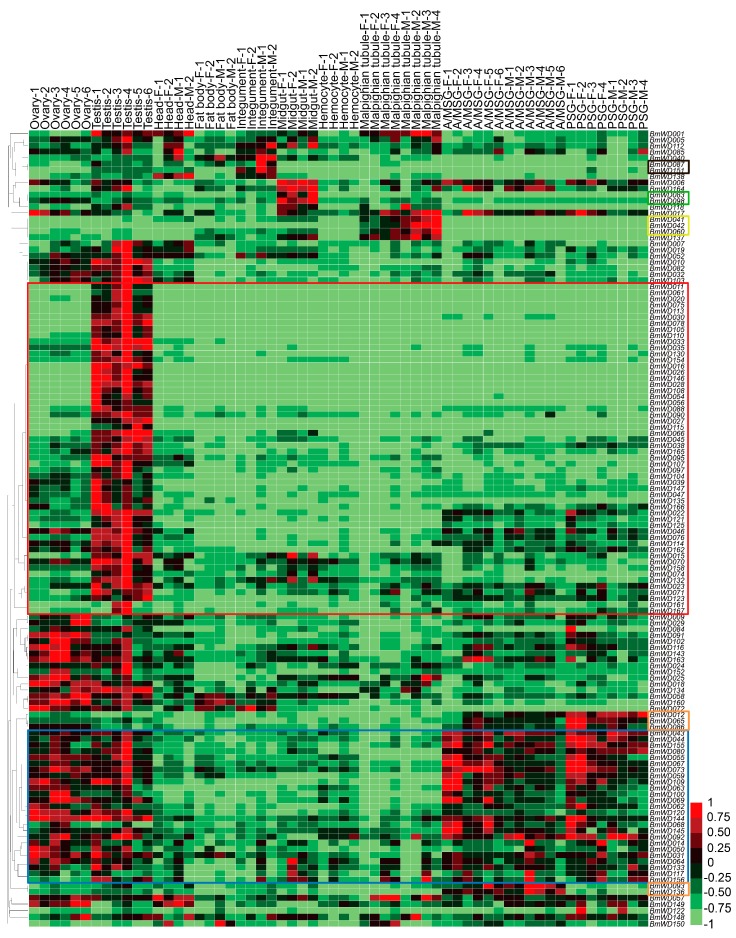
Microarray-based gene expression profiling of *BmWD40* genes in multiple tissues of silkworm larvae. The microarray gene expression dataset in multiple larval tissues of the silkworm on Day 3 of the fifth instar was downloaded from the SilkDB database. Repeated samples of each tissue are indicated with different numerals. A/MSG: Anterior/Median Silk Gland, PSG: Posterior Silk Gland, F: Female, M: Male. Red box indicates highly-expressed testis-specific genes. Blue box indicates the genes with high expression in gonad and silk glands. Orange boxes indicate the genes with silk-gland-specific high expression. Yellow box indicates genes that showed Malpighian-tubule-specific high expression. Green box indicates genes that were mainly expressed in the midgut. Black box indicates genes that were highly expressed in the integument.

A large number of *BmWD40s* showed tissue-specific gene expression features. Most *BmWD40s* showed high expression in the gonads, and a considerable proportion of these genes exhibited testis-specific high expression (red box in Figure 8); some of them were also highly expressed in silk glands (blue box in Figure 8). *BmWD012*, *BmWD065* and *BmWD086* were specifically highly expressed in the Posterior Silk Gland (PSG); *BmWD093* and *BmWD136* were highly expressed in the Anterior/Median Silk Gland (A/MSG). Furthermore, *BmWD041*, *BmWD042* and *BmWD060* exhibited Malpighian-tubule-specific high expression and showed higher expression in males. *BmWD083* and *BmWD098* were mainly expressed in the midgut. *BmWD087* and *BmWD151* were highly expressed in the integument (Figure 8). These diverse expression features indicate that certain *Bm*WD40 proteins participate in special physiological functions in specific tissues.

**Figure 9:**
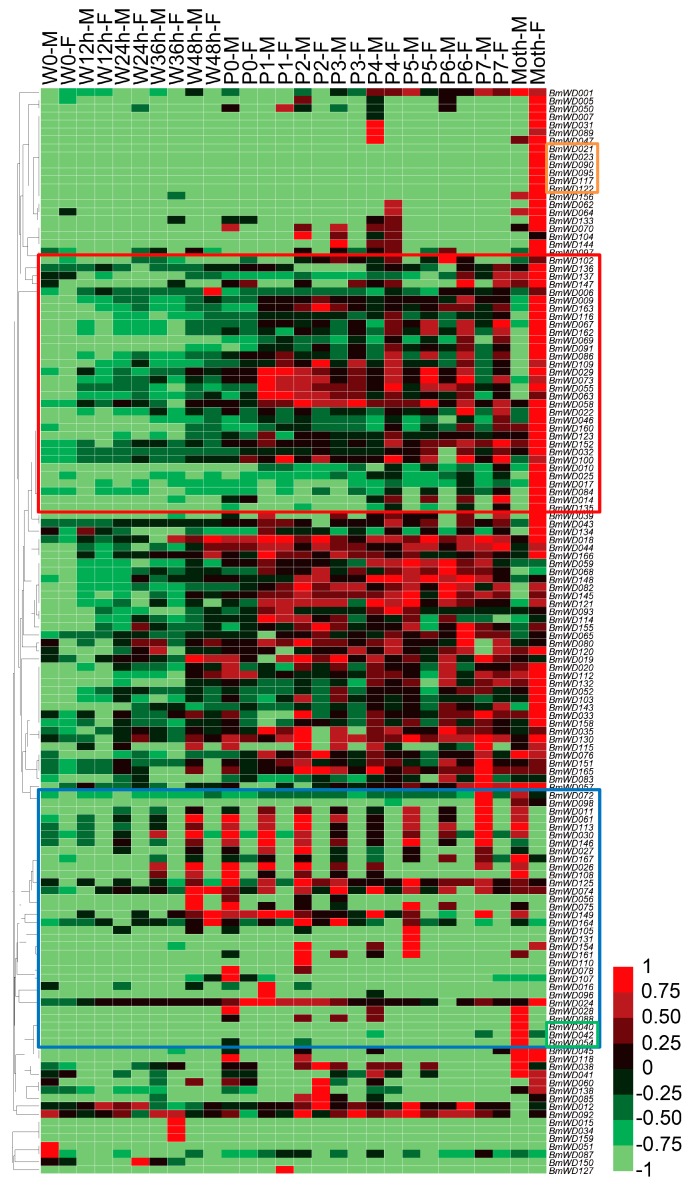
Microarray-based gene expression profiling of *BmWD40* genes during silkworm metamorphosis. One hundred thirty six *BmWD40* genes were detected to be expressed during silkworm metamorphosis. M: Male, F: Female, W: Wandering (W0: beginning of Wandering for spinning, W12 h: 12 h after Wandering), P: Pupation (P0: beginning of Pupation, P1: one day after Pupation), h: hour. Most genes in the red box were specifically highly expressed in female, whereas most genes in the blue box were male-specific. Genes in the green box exhibited male-moth-specific high expression. Genes in the orange box showed female-moth-specific expression.

We used the microarray gene expression data from late larval to adult stages of silkworm to study the temporal expression profiles of the *BmWD40* genes during metamorphosis. A total of 136 *BmWD40s* was found to be expressed during silkworm metamorphosis (Table S6). Most *BmWD40s* exhibited high expression in the pupal and adult stages (Figure 9), indicating that these genes were involved in silkworm metamorphosis. The hierarchical clustering graph showed that the expression pattern of many genes displayed sexual dimorphism (Figure 9). Most of the genes in the box outlined in red were specifically highly expressed in female, whereas the expression of most genes in the box outlined in blue were male-specific. Moreover, *BmWD040*, *BmWD042* and *BmWD054* exhibited male-moth-specific high expression and *BmWD021*, *BmWD023*, *BmWD090*, *BmWD095*, *BmWD117* and *BmWD122* exhibited female-moth-specific expression.

We found that many genes, such as *BmWD011*, *BmWD061*, *BmWD075* and *BmWD113*, were expressed specifically in the testis of larvae and in male individuals during metamorphosis (Table S6, Figure 8 and Figure 9), indicating that these genes may be required for testicular development or spermatogenesis. Among these, four genes, *BmWD016*, *BmWD105*, *BmWD113* and *BmWD161*, which belonged to Class 1 of the *BmWD40* family, encoded dynein intermediate chain proteins. The *dynein Intermediate Chain* (*IC*) genes are associated with sperm ultrastructure, and gene defects may bring about anomalies in the sperm flagella [53,54]. Accordingly, we propose that the spatiotemporally-specific expression of the four *IC* genes may be necessary for silkworm spermatogenesis or the formation and maintenance of spermatozoa flagella.

Our findings are also supported by functional studies of orthologs in other species. *WDR63*, *WDR78* [55] and *IFT140* [56], which are orthologs of the three highly-expressed testis-specific genes *BmWD027*, *BmWD054* and *BmWD104*, respectively, are also particularly highly expressed in the testis of mice, and they are related to sperm formation, sperm function and male fertility in mice. Knockout of *Pex7* [57] (ortholog of *BmWD132*, which is particularly highly expressed in the testis of silkworm) in mice leads to defects in plasmalogen biosynthesis and results in very-long-chain fatty acids-induced (VLCFA-induced) degeneration and apoptosis of spermatocytes. Furthermore, *WDR45* [29], *WDR47* [58], *WDR48* [59] and *GNB5* [60,61] (orthologs of *BmWD103*, *BmWD001*, *BmWD160* and *BmWD112*, which have relatively high expression in the heads of silkworm) are related to neurogenesis and brain development in humans, mice and nematodes. *FBXW7* [33] (ortholog of *BmWD005*, which is highly expressed in the midgut of silkworm) is involved in colorectal cancer. *WDR37* [62] (ortholog of *BmWD150*, which is highly expressed in the Malpighian tubules, an insect organ functionally homologous to mammalian kidneys) is associated with kidney function and chronic kidney disease.

### 2.6. Hotspot Residues of Protein-Protein Interactions

WD40 proteins can participate in protein-protein interactions with the help of certain major residues (hotspot residues) on their surfaces [9,11,63]. Therefore, we predicted potential hotspot residues on the *Bm*WD40 proteins using WDSP (http://wu.scbb.pkusz.edu.cn/wdsp/predictor.jsp). The results are shown in Table S1. To investigate the potential protein that interacts with WD40, we also analyzed protein-protein interaction networks based on the STRING database (Search Tool for Recurring Instances of Neighbouring Genes, http://string-db.org/), and proteins with potential interaction with the *Bm*WD40 proteins are listed in Table S7.

## 3. Materials and Methods

### 3.1. Genome-Wide Identification of WD40 Proteins

The whole-genome protein sequences of silkworm were downloaded from two silkworm genome databases, SilkDB (http://www.silkdb.org) and KAIKObase (http://sgp.dna.affrc.go.jp/KAIKObase/). The HMM profiles for the WD repeat (PF00400) were downloaded from the Pfam database (http://pfam.sanger.ac.uk/) [64]. The hmmsearch program (E-value ≤ 1 × 10^−1^, score ≥ 0) of the HMMER3.0 software (http://hmmer.org/) was used for the identification of WD40 proteins, and redundant sequences were discarded manually. The protein sequences of other species were downloaded from Ensembl (http://www.ensembl.org), and the same approach was used to identify WD40 proteins in 7 other insects: *Danaus plexippus* (Lepidoptera), *Heliconius melpomene* (Lepidoptera), *Anopheles gambiae* (Diptera), *Drosophila melanogaster* (Diptera), *Apis mellifera* (Hymenoptera), *Nasonia vitripennis* (Hymenoptera) and *Tribolium castaneum* (Coleoptera); as well as 9 vertebrates, including *Pan troglodytes* (Primates), *Mus musculus* (Rodentia), *Oryctolagus cuniculus* (Lagomorpha), *Sus scrofa* (Artiodactyla), *Ornithorhynchus anatinus* (Monotremata), *Gallus gallus* (Aves), *Anolis carolinensis* (Reptilia), *Danio rerio* (Cypriniformes) and *Latimeria chalumnae* (Coelacanthiformes); and 5 nematodes, including *Caenorhabditis elegans*, *Onchocerca volvulus*, *Brugia malayi*, *Pristionchus pacificus* and *Strongyloides ratti*.

The chromosomal locations, gene lengths, probes and predicted protein lengths were obtained from SilkDB and KAIKObase. Theoretical molecular weights and isoelectric point values were calculated online at http://web.expasy.org/protparam/. Three online programs, CDD (http://www.ncbi.nlm.nih.gov/Structure/cdd/wrpsb.cgi) [43], WDSP (http://wu.scbb.pkusz.edu.cn/wdsp/predictor.jsp) [45] and SMART (http://smart.embl-heidelberg.de/) [44], were used to confirm the WD40 repeats in the predicted proteins. The predicted WD40 protein genes were manually annotated by online BLASTP searches against the non-redundant protein database in NCBI (https://www.ncbi.nlm.nih.gov/).

### 3.2. Chromosomal Distribution and Gene Structure

Based on the chromosomal coordinates extracted from the KAIKObase, the WD40 genes were plotted onto chromosomes according to their order of physical position, and the “*BmWD40* distribution map” was displayed by MapChart [65]. The adjacent genes, which belonged to the same subfamily and were located within the same or neighboring genomic region, were regarded as tandem repeats [66]. To identify the *BmWD40s* related to potential segmental duplication events, we checked all the genes in the silkworm segmental duplication dataset of Zhao’s previous report [67], which contains the composition of genes in the silkworm segmental duplication regions. The exon-intron structures of the genes were determined by comparing protein coding sequences of *BmWD40s* with their corresponding genomic sequence.

### 3.3. Phylogenetic Analysis and Domain Architecture Annotation

The complete amino acid sequences or WD40 domain sequences of the identified *Bm*WD40 proteins were imported into MEGA6 [68], and multiple sequence alignments were carried out by the MUSCLE program [69]. The alignment result was then subjected to build an unrooted phylogenetic tree by the neighbor-joining method [51] with a bootstrap of 1000 replicates. Three online programs, WDSP [45], CDD [43] and SMART [44], were used to determine the domain architectures of the silkworm WD40 proteins.

### 3.4. Gene Ontology Annotation and Pathway Enrichment

Gene Ontology (GO) analysis was performed using Blast2GO software (Version 4.0.7, BioBam Bioinformatics S.L., Valencia, Spain) with default settings [70]. GO annotation of genes was also obtained from the two silkworm genome databases (SilkDB and KAIKObase). KOBAS v2.0 (http://kobas.cbi.pku.edu.cn) [71] was used to test the statistical enrichment of *BmWD40* genes in Kyoto Encyclopedia of Genes and Genomes (KEGG) pathways. KEGG pathways with a corrected *p*-value less than 0.05 were considered as enriched.

### 3.5. Expression Profiling Using Microarray Data

To elucidate the tissue-specific expression profile of *BmWD40* genes, the microarray gene expression data of 10 larval tissues on Day 3 of the fifth instar, namely head, ovary, testis, fat body, integument, midgut, Malpighian tubule, hemocyte, Anterior/Middle Silk Gland (A/MSG) and Posterior Silk Gland (PSG), were retrieved from SilkDB (http://www.silkdb.org). Each tissue sample was analyzed using at least two biological repeats. The microarray data were analyzed using a previously-described protocol [72]. Raw microarray data were normalized using a linear normalization method, in which four confirmed house-keeping genes (encoding proteasome β subunit, eIF 4A, eIF 3A subunit 5 and eIF-3 subunit 4) were used. The normalized values were analyzed using one-way Analysis Of Variance (ANOVA) across all investigated tissues (*p* < 0.001). The filtered data were used to examine differential gene expression with log2 (Fold-change) of ±1 and *p* < 0.01 (unpaired *t*-tests). Genes with normalized signal intensity values less than 400 were defined as “unexpressed”. 

To elucidate the expression pattern of *BmWD40* genes during silkworm metamorphosis, we analyzed the microarray gene expression data at 14 developmental time points from late larval to adult stages, including the spinning stage (beginning of Wandering for spinning (W0), 12 h after Wandering (W12 h), W24 h, W36 h and W48 h), pupal stage (beginning of Pupation (P0), 1 day after Pupation (P1), P2, P3, P4, P5, P6 and P7) and adult. Each sample was analyzed using two biological repeats. Microarray hybridization was performed as previously reported [72], and the microarray data were processed as described in a previous report [73]. Briefly, gene expression on Day 3 of the fifth Larval instar (L5D3) was used as a control. If the signal intensity of a gene exceeded 400 units at a time point, the gene was considered to be expressed. Then, the ratio of the signal intensities of each *BmWD40* gene at each developmental time point to that in the L5D3 control was used to detect dynamic changes in the expression levels.

The hierarchical clustering graph showing spatiotemporal expression profiles was generated based on average linkage clustering and a Pearson correlation distance metric using the Heat map Illustrator (HemI) software package (version 1.0.3.3, Huazhong University of Science and Technology, Wuhan, Hubei, China) [74].

## 4. Conclusions

WD40 proteins play key roles in dissimilar biochemical mechanisms and various biological processes. We conducted a genome-wide analysis and identified the numbers of WD40 protein genes in 9 vertebrates (mean ~256), 8 insects (mean ~178) and 5 nematodes (mean ~135). The WD40 protein family has undergone lineage-specific expansions in animal evolution. Sequence analyses of the silkworm suggested that tandem and segmental duplication did not play a major role in producing the current number of *BmWD40s* and that the gene duplication events occurred predominantly prior to the domain recombination events in the evolution of the multi-domain *BmWD40s*. Microarray-based expression profiling revealed the tissue-specific, stage-specific, or sex-dimorphic expressions of the *BmWD40* genes. These findings increase our understanding of the evolution of the WD40 protein family in animals and provide detailed knowledge of the silkworm WD40 protein family. The exact functions of these differentially-expressed *BmWD40* genes during silkworm development require future characterization.

## Supplementary Materials

Supplementary materials can be found at http://www.mdpi.com/1422-0067/19/2/527/s1.

## Abbreviations

WD	Tryptophan-Aspartic Acid (W-D)
LisH	Lis Homology
BEACH	Beige and Chediak–Higashi
CAF-1	Chromatin Assembly Factor-1
VLCFA	Very-Long-Chain Fatty Acids
