# Supplementary material for: Genome-Wide Identification and Characterization of WD40 Protein Genes in the Silkworm, Bombyx mori
Source: Int J Mol Sci. 2018 Feb 9;19(2):527. doi: 10.3390/ijms19020527 (PMC5855749; doi:10.3390/ijms19020527)
Supplement: Supplementary file 1 [file ijms-19-00527-s001.zip › ijms-260156-supp-proofreading/ijms-260156-Supplementary Materials.docx]

**Supplementary Materials**

**
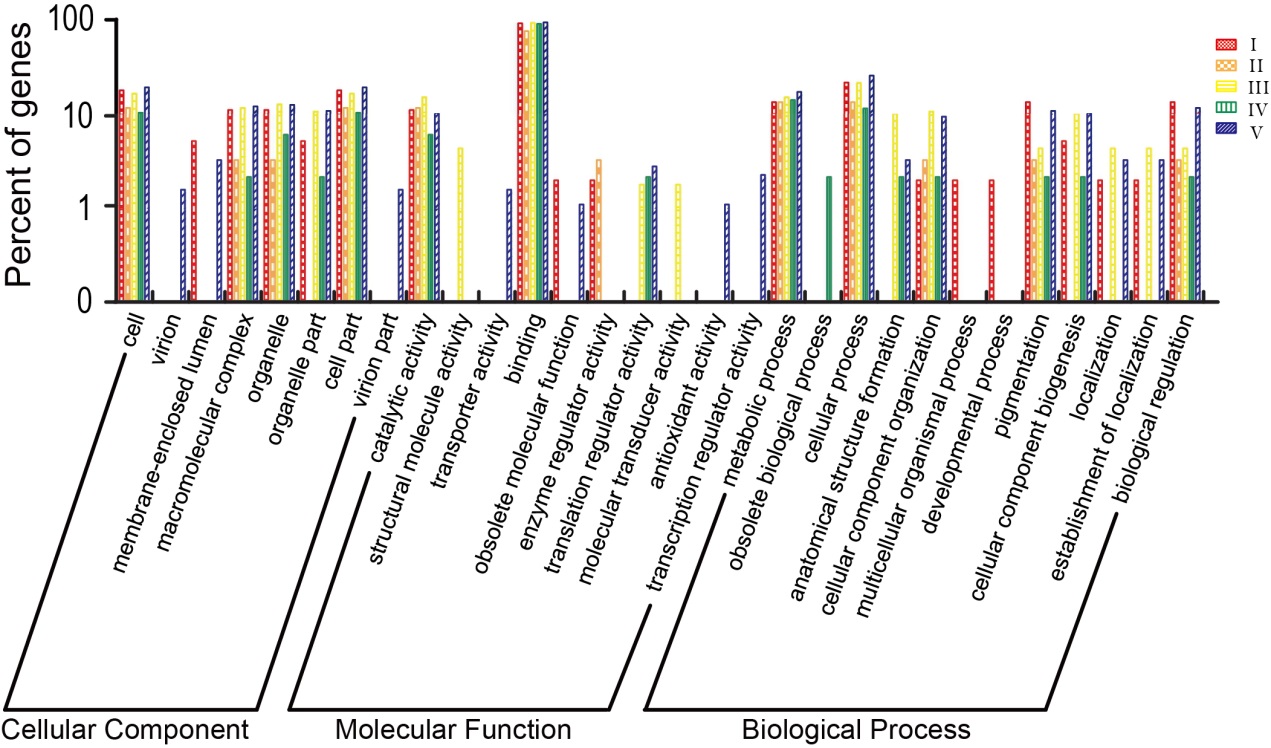
**

**Figure S1.** GO categories of *BmWD40* genes from the five clusters, respectively. The five clusters (I–V) are indicated by red, orange, yellow, green and blue, respectively.


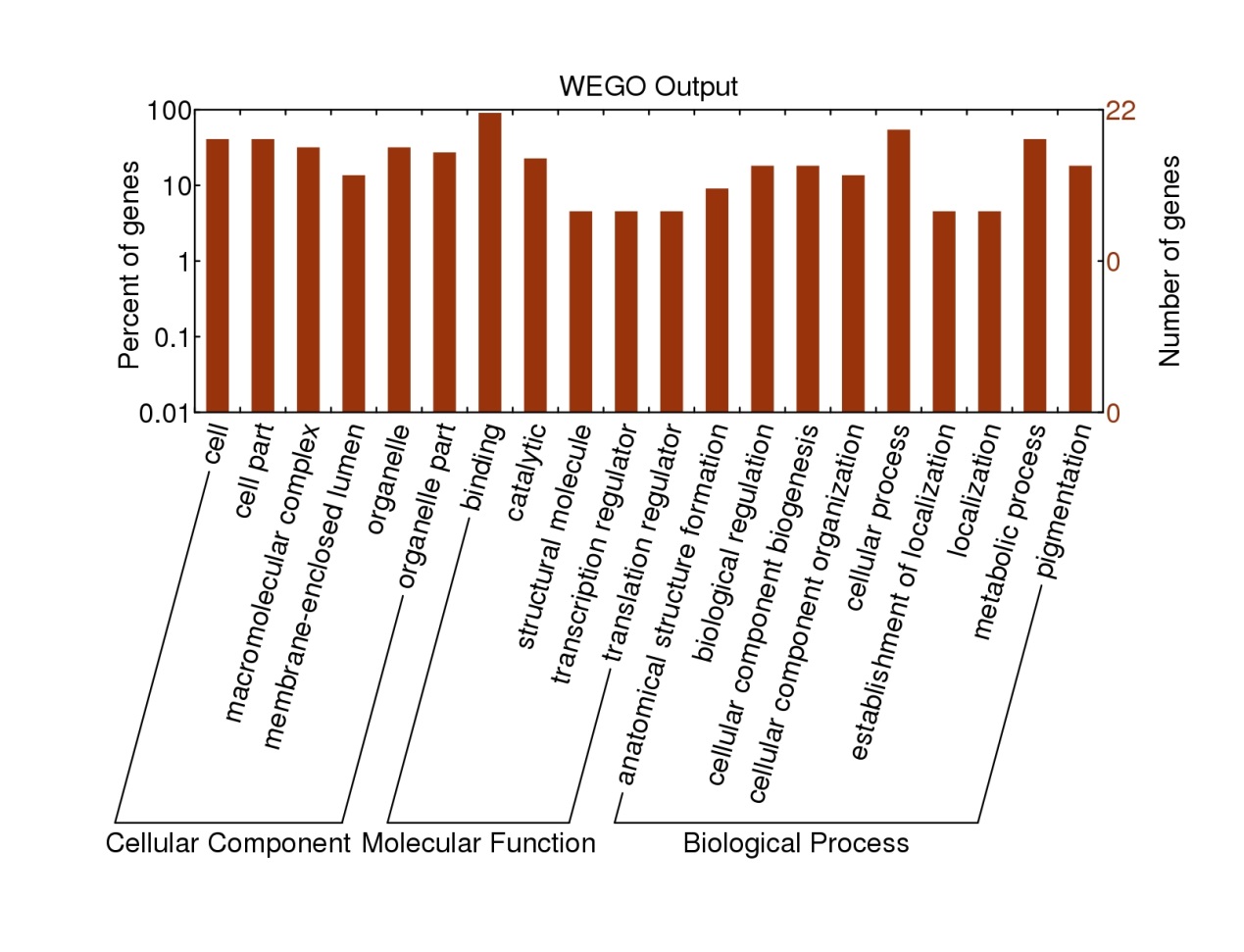


**Figure S2.** GO categories of the 22 *BmWD40* genes expressed in all investigated tissues. This analysis was visualized with WEGO. Details are provided in Supplement File Table S5.

**Table S1.** Summary of 172 *BmWD40* protein genes.

**Table S2.** A catalog of WD40 protein genes in 7 other insects, as well as 9 vertebrates and 5 nematodes.

**Table S3.** Gene Ontology (GO) classification of the *BmWD40s*.

**Table S4.** KEGG classification of the *BmWD40s*.

**Table S5.** Microarray gene expression data of *BmWD40s* in multiple tissues of silkworm larvae.

**Table S6.** Microarray gene expression data of *BmWD40s* during metamorphosis.

**Table S7.** List of proteins with potential interaction with the *Bm*WD40 proteins.
