# Supplementary figures and images for: Genome-Wide Identification and Characterization of WD40 Protein Genes in the Silkworm, Bombyx mori
Source: Int J Mol Sci. 2018 Feb 9;19(2):527. doi: 10.3390/ijms19020527 (PMC5855749; doi:10.3390/ijms19020527)

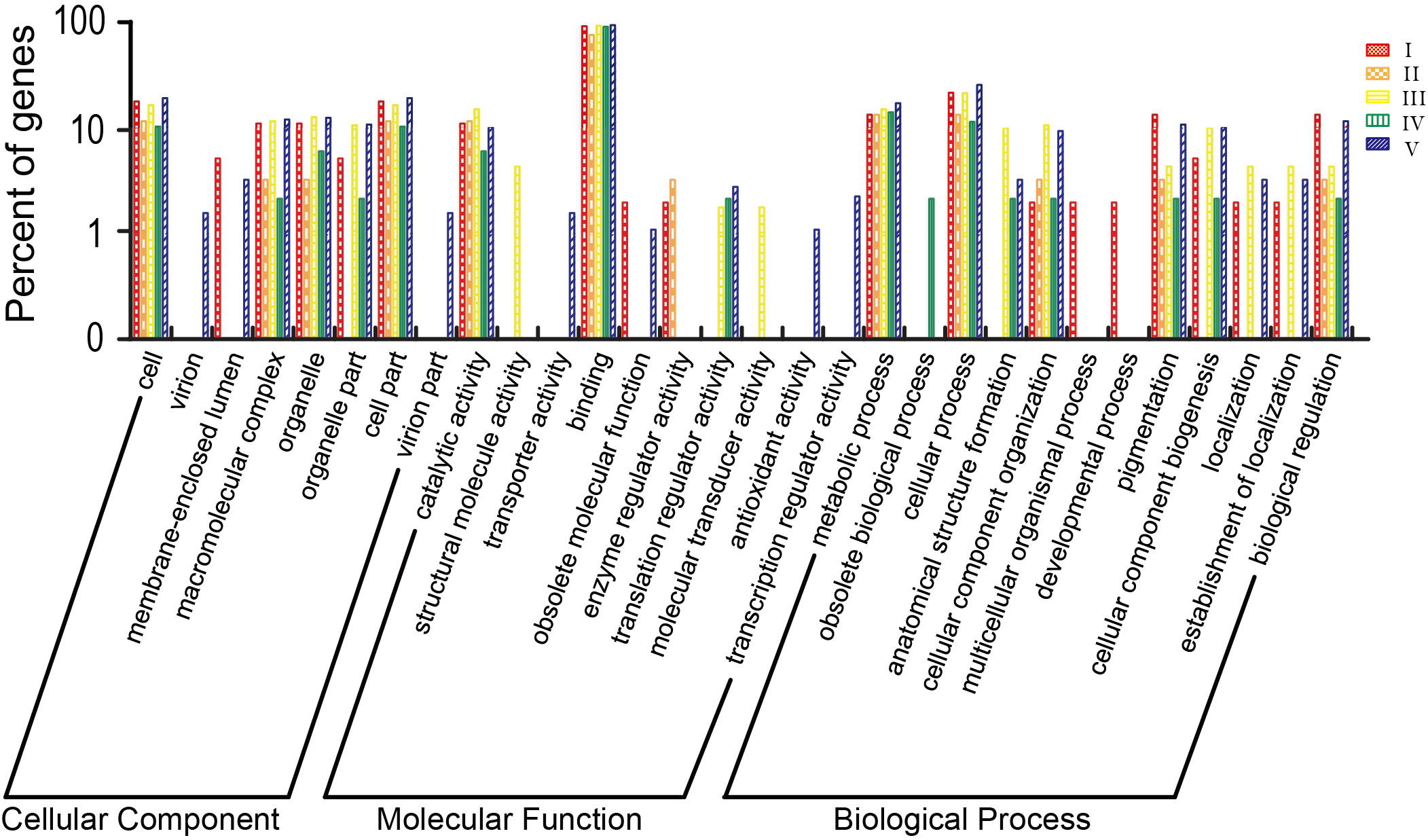

Supplement: Supplementary file 1 [file ijms-19-00527-s001.zip › ijms-260156-supp-proofreading/Figure S1.jpg]

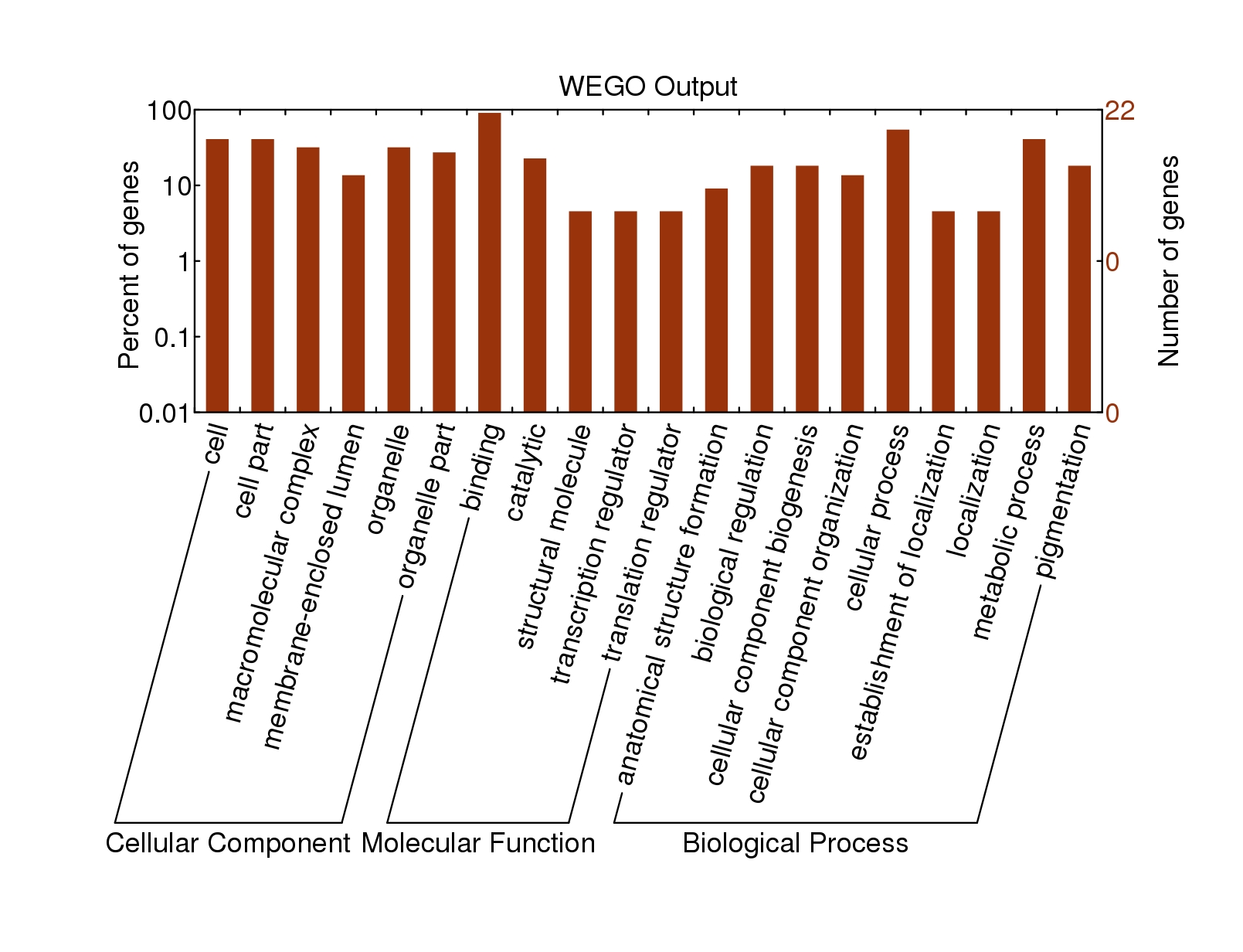

Supplement: Supplementary file 1 [file ijms-19-00527-s001.zip › ijms-260156-supp-proofreading/Figure S2.jpg]
